# Infarctus de l´os fémoral révélant une drépanocytose composite SC chez un patient marocain

**DOI:** 10.11604/pamj.2020.36.361.22688

**Published:** 2020-08-28

**Authors:** Fatima-Zahrae Bennis, Asmae Biaz, Aida Zkik, Achraf Rachid, Sanae Bouhsain, Abdellah Dami, Elmachtani Idrissi Samira

**Affiliations:** 1 Laboratoire de Biochimie-Toxicologie de l´Hôpital Militaire d´Instruction Mohammed V, Rabat, Maroc,; 2 Faculté de Médecine et de Pharmacie, Université Mohammed V, Rabat, Maroc

**Keywords:** Hémoglobinopathie, drépanocytose composite SC, infarctus osseux, dépistage précoce, Hemoglobinopathy, compound heterozygous SC sickle cell disease, bone infarction, early detection

## Abstract

La double hétérozygotie SC est considérée comme un syndrome drépanocytaire majeur; en effet, son évolution peut être marquée par des complications sévères voire irréversibles, tel que l´infarctus osseux. Notre observation rapporte la découverte d´une hétérozygotie composite SC chez un patient de 17 ans à la suite de gonalgies intenses et met ainsi en exergue le retard diagnostic de cette maladie, et soulève la nécessité de mise en place d´une politique de dépistage précoce afin d´améliorer la prise en charge et le pronostic des sujets atteints.

## Abstract (en)

Double heterozygosity SC is a major sickle cell syndrome. Its course may be marked by severe or irreversible complications, such as bone infarction. We here report the case of a 17-year-old patient presenting with compound heterozygous SC sickle cell disease following severe knee pain (gonalgia) in order to highlight the risk of delayed diagnosis as well as the need for prevention policies guiding early screening, thus improving patients’ management and prognosis.

## Introduction

Les syndromes drépanocytaires majeurs (SDM) sont des maladies génétiques de transmission autosomique récessive. L´état homozygote SS est la forme la plus fréquente de ces affections, mais d´autres allèles des gènes ß de l´hémoglobine (Hb) peuvent s´associer à l´HbS et induire un SDM [1]; dont la drépanocytose hétérozygote composite SC qui peut représenter jusqu´à 20 à 30% des SDM [2]. Celle-ci présente un tableau clinico-biologique plus discret que celui de la drépanocytose SS, ce qui est à l´origine d´un retard diagnostic trop souvent au stade de complications irréversibles [3]. Nous rapportons le cas d´une drépanocytose hétérozygote composite découverte à la suite d´un infarctus osseux du membre inférieur.

## Patient et observation

**Figure 1:**
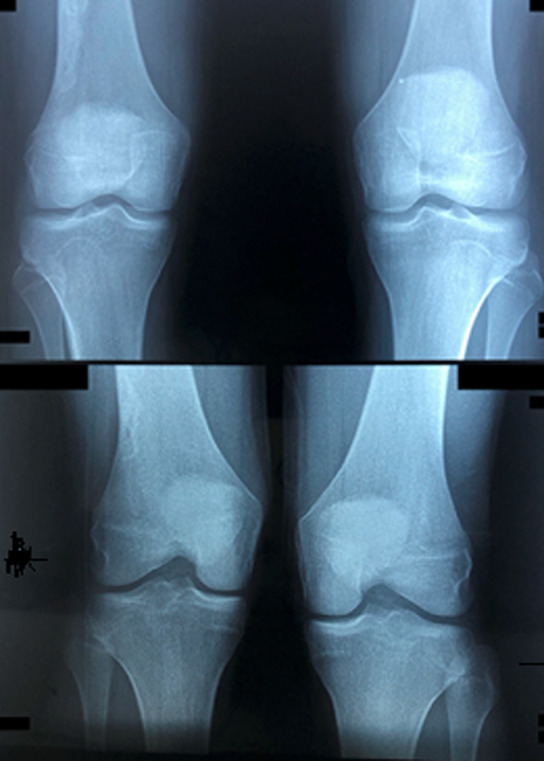
radiographie standard des genoux (de face)

**Figure 2:**
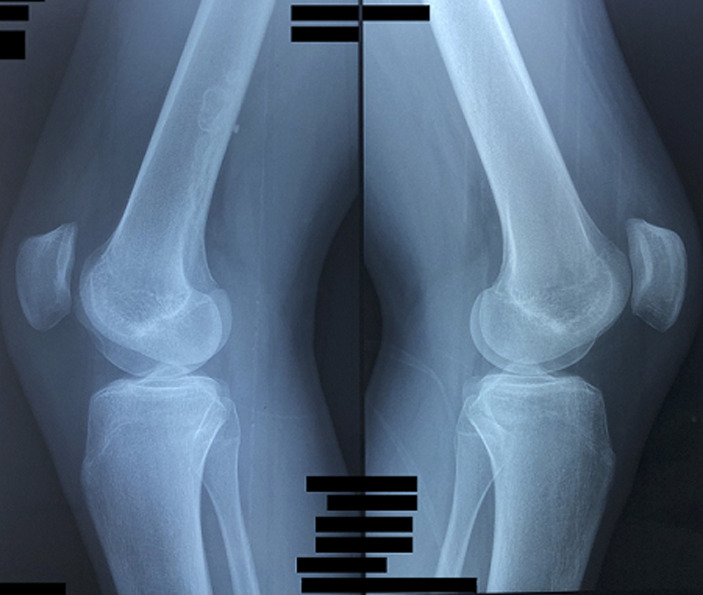
radiographie standard des genoux (de profil)

Il s'agit d'un patient âgé de 17 ans, célibataire, d´origine marocaine, sans antécédents particuliers notables, hospitalisé au service de rhumatologie pour gonalgies intenses. Le début de la symptomatologie remonte à un mois avant son hospitalisation par l´installation brutale de gonalgies gauches antérieures intenses (EVA = 7/10) permanentes et rebelles au traitement antalgique usuel, avec migration de la douleur vers le côté controlatéral. L´examen clinique retrouve un syndrome rotulien bilatéral. A l´imagerie, la radiographie standard du genou droit face et profil révèle une image évoquant un infarctus osseux au niveau de la diaphyse fémorale droite (Figure 1, Figure 2).

**Figure 3:**
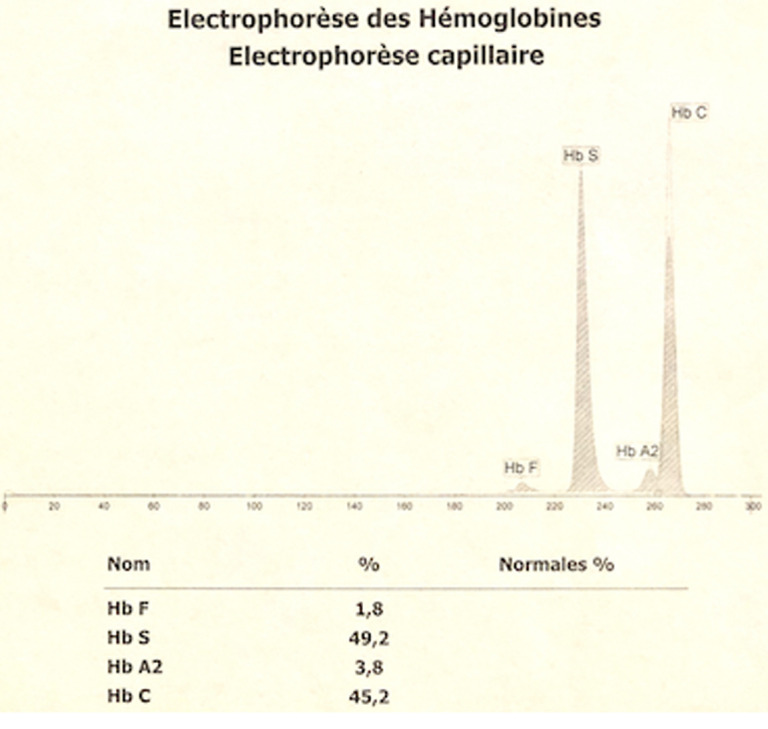
profil électrophorétique de l´hémoglobine à pH alcalin

**Figure 4:**
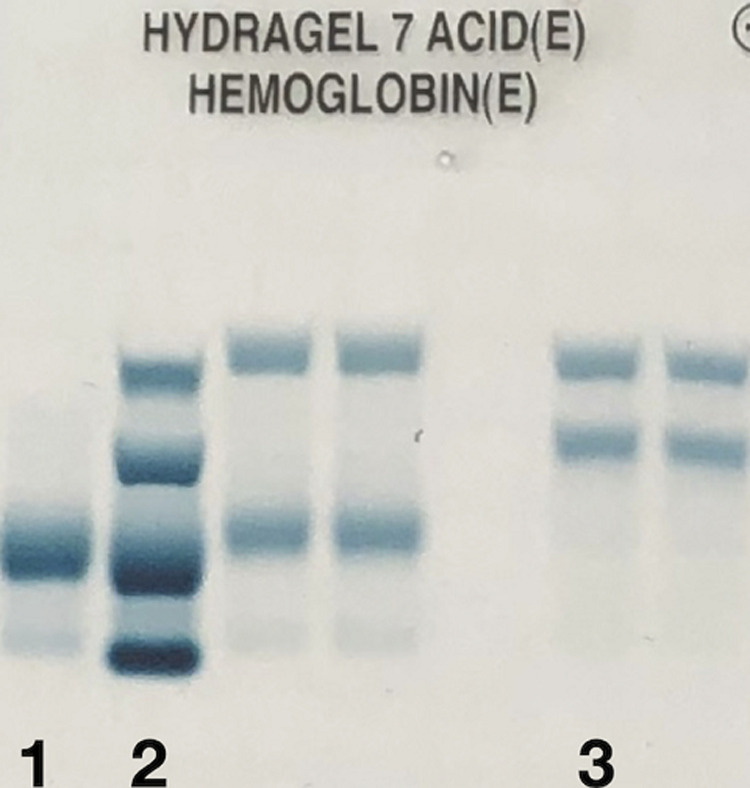
électrophorèse de l´hémoglobine à pH acide; 1) contrôle normal ; 2) contrôle pathologique AFSC ; 3) patient

Au bilan biologique, l´hémogramme objective une discrète anémie avec un taux d'Hb à 11,8 g/dL, hypochrome microcytaire (volume globulaire moyen (VGM) à 71,8 fl et teneur corpusculaire moyenne en hémoglobine (TCMH) à 25,8 pg). Le dosage de la bilirubine totale (BT) et directe (BD) est en faveur d´une hémolyse avec une BT à 21 mg/l et une BD à 6 mg/l. Le bilan inflammatoire à la recherche d´une étiologie infectieuse est négatif avec une CRP à 3,7 mg/l, une VS à 5mm à la première heure, un profil électrophorétique des protéines sériques normal et un taux de polynucléaires neutrophiles à 5700/mm^3^. Une électrophorèse capillaire de l'hémoglobine à pH alcalin est réalisée sur le système Capillarys 2 Flex piercing (Sebia®). Elle a révélé l'absence d'HbA, la présence d'Hb S à 49,2%, Hb C à 45,2%, HbA2 à 3,8% et Hb F à 1,8% (Figure 3). L'électrophorèse de l'hémoglobine à pH acide sur gel d'agarose sur l'automate Hydrasys 2 Scan (Sebia®) confirme la double hétérozygotie SC (Figure 4).

Devant le tableau clinique, radiologique et biologique, le diagnostic retenu est un infarctus osseux compliquant une drépanocytose hétérozygote composite SC chez un patient de 17 ans. Le traitement est basé sur la prescription d´antalgiques, d´anti-inflammatoires non stéroïdiens et d´une mise en décharge, ainsi que d´une consultation spécialisée au service d'Hématologie Clinique en vue d'une prise en charge pluridisciplinaire.

## Discussion

L'OMS estime le taux des porteurs d´une hémoglobinopathie à 6.5% de la population mondiale [4]. Quant à la drépanocytose, elle est probablement la maladie génétique la plus fréquente au monde, que les flux migratoires ont rendu maintenant ubiquitaire; et faisant de cette affection un véritable problème mondial de santé publique car en effet, chaque année plus de 500 000 enfants drépanocytaires naissent, dont 300 000 en Afrique dont plus de la moitié meure avant l´âge de 5ans [4]. Une étude rétrospective sur une période de 10 ans réalisée au sein de notre formation a recruté 42 cas de drépanocytose dont 32 cas de drépanocytose hétérozygote A/S, 6 cas de drépanocytose S/S et 4 cas de drépanocytose SC; la double hétérozygotie représentant ainsi 9,52% des cas de drépanocytose [5]. La physiopathologie de la drépanocytose résulte d´une mutation ponctuelle portant sur le sixième codon du gène β-globine, substituant l´acide glutamique « hydrophile » par une valine « hydrophobe » donnant ainsi un gène β muté codant pour l´HbS qui peut établir des liaisons hydrophobes avec la chaine β d´une autre molécule d´Hb. Cette polymérisation en fibres hélicoïdales et leur rigidification engendrent la déformation des globules rouges (GR) en faucilles. Cette falciformation est à l´origine des phénomènes vaso-occlusifs, et est favorisée par plusieurs facteurs dont la déshydratation cellulaire [6]. Or, la présence d´HbC dans le GR est associée à la déshydratation de ce dernier par l´activation de la pompe K-Cl, et favorise donc sa déformation [7]. Par ailleurs, l´augmentation de la température serait aussi un facteur amplifiant le phénomène de falciformation [6]; notre patient ayant rapporté la notion de « hammam » juste avant le début de la symptomatologie.

Parmi les signes cliniques décrits chez les patients drépanocytaires, certains sont classiques de la petite enfance et font d´emblée évoquer le diagnostic; il s´agit notamment de la dactylite. En revanche, d´autres signes sont moins spécifiques et entrent dans le cadre d´une pathologie générale pouvant conduire à un retard de diagnostic: c´est le cas des patients HbSC qui présentent habituellement un syndrome drépanocytaire modéré [8]. Comme en témoigne une étude réalisée à Chicago portant sur 106 cas de drépanocytose type SC, 54 patients soit plus de la moitié des cas n´ont été diagnostiqués qu´après l´âge de 18ans [9]. Chez notre patient, le diagnostic n´a été posé qu´à l´âge de 17 ans et seule une discrète anémie a été retrouvée à l´hémogramme, sans notion de syndrome anémique ou de notion de transfusion antérieure. L´hémolyse chronique chez les drépanocytaires est responsable de l´anémie. Celle-ci, bien qu´également présente chez les patients HbSC, y est plus souvent modérée, habituellement comprise entre 100 g d´Hb/l et 120 g d´Hb/l; ce qui participe également à la discrétion de ce type de SDM, et donc au retard diagnostic. A ce propos, une étude comparative réalisée au Burkina Faso portant sur 61 cas de drépanocytose dont 38 cas de drépanocytose type HbSC et 23 cas de type HbSS, l´anémie sévère était présente chez 10 % des drépanocytaires type HbSC versus 26% chez ceux du type HbSS de même les transfusions sanguines qui ont été nécessaires chez seulement 16% des sujets HbSC contre 43% des sujets HbSS [7].

Bien que la symptomatologie soit en général un peu moins sévère, l´évolution de la drépanocytose type HbSC est marquée par des complications chroniques, dominées par les problèmes orthopédiques et ophtalmologiques entrainant parfois des séquelles irréversibles [10]. Ceci peut être illustré par une étude réalisée en Jamaïque qui a montré que, sur 89 cas de drépanocytose type HbSC, 33% des cas ont présenté une rétinopathie proliférative, 12% une ostéonécrose aseptique de la tête fémorale, alors que les complications biliaires, l´hématurie, l´hypertension artérielle pulmonaire, et les infarctus spléniques n´ont pas été retrouvés [10]. Chez notre patient, le diagnostic n´a été posé qu´à la suite de gonalgies intenses, rebelles et invalidantes probablement en rapport avec un infarctus osseux. On peut conclure qu´en l´absence d´un dépistage néonatal, le diagnostic est posé trop tardivement. Pourtant un diagnostic précoce de la maladie permet d´obtenir une diminution significative du taux de mortalité et du nombre de complications [11]. Ce retard pourrait donc être évité par la mise en place d´un programme national de lutte contre la drépanocytose comprenant un dépistage néonatal systématique, ou du moins un dépistage prénuptial voire pré-implantatoire. Il constituerait le premier pas d´un trajet de soins spécifiques et adaptés pour les enfants atteints d´un SDM.

## Conclusion

Notre observation met en exergue le retard diagnostic des sujets ayant un SDM type hétérozygote composite SC, dont l´évolution est marquée par des complications irréversibles. Consciente de cette situation, l'OMS a déclaré dans plusieurs assemblées, l'urgence pour les pays touchés dont le Maroc de concevoir et de mettre en œuvre des programmes nationaux intégrés de prévention et de prise en charge de la drépanocytose et des autres hémoglobinopathies.
